# Alpha Oscillations in Parietal and Parietooccipital Explaining How Boredom Matters Prospective Memory

**DOI:** 10.3389/fnins.2022.789031

**Published:** 2022-04-13

**Authors:** Pin-Hsuan Chen, Pei-Luen Patrick Rau

**Affiliations:** Department of Industrial Engineering, Tsinghua University, Beijing, China

**Keywords:** electroencephalography, boredom, prospective memory, parietooccipital, parietal

## Abstract

Intelligent interaction alters previous human–machine task allocation patterns. Human workers will suffer from boredom and inattention, posing a significant challenge for the human–machine interaction loop. This study aims to investigate the relationship between boredom and prospective memory, which is a memory form including the detecting, identifying, and executing functions. Thus, the attention and memory mechanisms are critical to complete prospective memory tasks when bored. This study recruited twenty-eight participants and used electroencephalography to measure the alpha power in brain regions. The results indicated that parietal oscillations had a mediation effect on prospective memory, which could be associated with the frequent unstable attention. In addition, this study found that parietooccipital oscillations linked boredom and prospective memory, and the default mode network (DMN) and visual processing during boredom could better explain this finding. The findings of this study suggested that attention management and influences of processing visual information were starting points to cope with boredom because they could help prepare for prospective memory and make optimal decisions accordingly.

## Introduction

The growing intelligence and autonomy of systems extend human capabilities but challenge human ability to oversee and interact with systems effectively when needed. Interacting with intelligent and autonomous systems increases passive user interaction, and human workers are just like supervisory controllers. Thus, human workers are easily getting bored because they are experiencing long idle time and low task load in those passive user interaction tasks, such as monitoring and vigilance.

Boredom could decline task performances due to the failure of attention management in passive user interaction. In most cases, humans are expected to keep attention and vigilance to deal with occasional but urgent tasks through their event-based or time-based cues. However, those who are bored and inattentive are likely to miss cues and fail to complete tasks. In workplaces such as aviation and medicine, workers are well-training to execute future works with cues associated with their prospective remembering of intentions (Key Dismukes, 2012). Theoretically, prospective memory is an ability not only to ensure human implementation in future situations, but also to return to the interrupted task receiving cues and entering memory retrieval procedures (Dodhia and Key Dismukes, 2008). According to the dynamic multiprocess framework, detecting cues related to previous training is vital to initiate prospective memory retrieval (Scullin et al., 2013; Shelton and Scullin, 2017). Whereas both boredom and attention failures could hinder the initiation of prospective memory retrieval and increase vulnerability to forgetting. It is worth noticing that passive user interaction is universal in dull but intelligent workplaces where humans are vulnerable to boredom and forgetting intentions. Thus, preventing humans from prospective memory impairment in these boring situations is crucial.

Boredom is a transient and complex emotional state that occurs when someone is unable to reach an exact positive or negative emotion (Bench and Lench, 2019). Attention failures are often used to describe boredom because, in most boring conditions, people find it hard to concentrate on internal or external facts (Eastwood et al., 2012). In other words, boredom is related to unfavorable experiences. Specifically, a boring situation features a lack of engagement and interest, weak meaningfulness, and monotonous or low stimulation (Milyavskaya et al., 2019; Westgate, 2020).

The enhancement of machine intelligence greatly reduces task load, task engagement, and meaningfulness. Human workers are difficult to focus on their tasks. They seek compensated measures to relieve the adverse effects of inattention and boredom, namely, mind-wandering, daydreaming, distraction, and seeking. Notably, these measures are highly related to attention management. For example, mind-wandering and daydreaming shrink part of the attention resources, distraction diverts attention away from the ongoing task, and the seeking state might directly change one’s feelings and thoughts (John et al., 2005; Smallwood and Schooler, 2006; Matthews et al., 2010; Cummings et al., 2015; Daniels et al., 2015). Thus, the measures to cope with boredom pose a significant challenge for human workers while interacting and allocating tasks with intelligent systems.

Different coping strategies of boredom could impair prospective memory in various ways. First, distraction is associated with frequent attention shifting. People could not manage their attention effectively to focus on cue detection. In addition, detecting irregular and less focal cues is challenging because it requires a higher demand of attentional resources (Rose et al., 2010; Cummings et al., 2013). Poor attention management could hinder prospective memory. Second, mind-wandering is negatively related to working memory span, while the higher the working memory span, the better the prospective memory (Kane et al., 2007; Smith et al., 2011). That is, working memory span might be narrowed while mind-wandering, and then prospective memory performance is declined. Seli et al. (2018) focused on the reaction time of prospective memory and reported that a more extended time was required while mind-wandering. However, prospective memory is a continual mechanism; the reaction time for detecting and identifying cues is too short to successfully retrieve memory when people are facing attentional failures and shrinking working memory span during boredom (Fish et al., 2010; Hainselin et al., 2011; Yang et al., 2015). Therefore, based on previous literature, this study argues the adverse impact of boredom on prospective memory.

Neuroscience researchers have found that although the relationship between alpha power and different arousal levels could represent diverse brain functional states, boredom is an emotion related to alpha activity happening in both low- and high-arousal situations (Fahlman et al., 2013). Drowsiness, a low arousal emotion, weakens the alpha coherence of brain regions, which indicates an increase in internal attention and a decrease in vigilance level (Cantero et al., 1999). In contrast, high-arousal emotion strengthens the alpha coherence of brain regions and might imply external attention and distraction. Several electroencephalography (EEG) studies further indicated the activity of different brain regions could indicate different boredom coping strategies, for example, sensation-seeking was positively correlated with the left frontal activity (Santesso et al., 2008). In addition, Basharpoor et al. (2019) revealed that the left frontal activity was highly associated with execution and cognitive control. Moreover, the alpha power in the left and right frontal lobes has been found to show one’s attentional status, which would increase when one experienced a low task load (Klimesch, 1999). The intensity in the right middle temporal gyrus is highly associated with situational and environmental contexts and, thus, helps people concentrate on the ongoing task and avoid missing out (Lai et al., 2016). Furthermore, previous studies have found that the dynamic oscillations resulting from dorsal and ventral attention networks indicated the attentional shifting and reorienting (Corbetta et al., 2008; Vossel et al., 2014; Proskovec et al., 2018). The simultaneous function of dorsal and ventral attention networks leads to the junction of activity in different brain regions, for example, the bilateral parietotemporal (PT) junction (Vossel et al., 2009).

Neurocorrelated evidence of attention on prospective memory indicates that people draw attention away from the ongoing task while detecting the cues of prospective memory (Wang et al., 2013). According to the results from functional MRI (fMRI), the temporal and occipital cortexes are involved in the cue detection process (Reynolds et al., 2009; Peira et al., 2016). In addition, the whole perspective memory task is associated with the dorsolateral and inferior prefrontal cortex, inferior parietal cortex, precuneus, and anterior cingulate cortex (Okuda et al., 2007; Bisiacchi et al., 2011; Burgess et al., 2011; Wang et al., 2013). Although researchers have reported activation differences in the left and right frontal lobes concerning the event-based and time-based prospective memory based on MRI, their major findings focused on the great relevance of frontal lobe and prospective memory (Okuda et al., 2007).

Studies on boredom have mainly concentrated on causes and coping strategies, and few have investigated how boredom influenced human jobs and behaviors, such as prospective memory. Forgetting tasks in work settings, namely, healthcare and air traffic control, can result in severe consequences (Key Dismukes, 2012; Grundgeiger et al., 2014). This study focuses on the environment, which is of low task load and highly intelligent, and investigates human behavior with neuroscience evidence. Based on the assumption that boredom could decline the prospective memory performances, this study expects to figure out which boredom coping strategies are more likely to impair prospective memory during the task by observing alpha power in different brain regions. Although prospective memory impairment resulting from boredom could be associated with multiple boredom coping strategies, this study proposes research question 1 to identify the most critical coping strategy. Thus, this study could clarify which coping strategy in the studied environment impairs prospective memory and should be mitigated in the future. Moreover, this study is interested in predicting prospective memory while people are bored. The prediction model could further reflect the specific brain activity for effectively predicting the prospective memory performance. With the model, this study pinpoints the brain region enhancing the predictability of prospective memory while boredom. This way, the activity of the important brain region could help reveal the effectiveness of boredom intervention in the future. This study then proposes research question 2 to specify the alpha power in significant brain regions in the studied environment.

Research question 1: What boredom coping strategy could negatively influence the prospective memory?

Research question 2: Based on alpha power, what are the significant brain regions for predicting prospective memory while boredom?

This study applies EEG to measure the differences in alpha power while conducting prospective memory tasks since boredom is often associated with brain activity in the alpha band. Hence, this study analyzes alpha power based on the neural oscillations in the frontal, parietal, temporal, and occipital regions.

Human–machine task allocation is different from prior experiences because of the increasingly passive user interaction. Besides, previous patterns and issues of human–machine interaction no longer fit future models. Consequently, alleviating and avoiding boredom, which might result in undesirable outcomes, such as poor decision-making due inattention, is critical. The results of this study may help to determine the relationship between boredom and prospective memory. Moreover, this study could be the foundation to develop boredom intervention methods and provide suggestions from a neuroscience perspective for human-centered intelligent system design.

## Materials and Methods

### Participants

This study recruited 31 university students. Three participants were excluded from the analysis, two of them had noisy and unusable EEG data, and the other had invalid memory responses. Overall, there were 28 valid samples included in this study. The number of males and females was equal, and the age ranged from 19 to 27 (mean = 22.89, *SD* = 2.15). All of them were healthy, had normal or corrected-to-normal vision, and had good mental health.

### Procedure

Upon obtaining informed consent, the experimenter prepared a 64-electrode EEG montage. Then, the experimenter presented the experiment instructions, namely, participants’ responsibilities, the structures and functions of the experimental dashboard, and the prospective memory task procedure. Participants were asked to play as homecare nurses monitoring and operating multidimensional homecare items through a smart homecare control dashboard to support patients. After that, participants were guided to operate the system for the exercise. A prospective memory task was given after ensuring that the participant understood how to interact with the control dashboard.

The prospective memory task in this study was designed with three sequential phases based on Glienke and Piefke (2016), namely, planning (memorizing future intentions based on either time or events), retention (interval between memory encoding and retrieval), and performance (execution with memory). This study considered prospective memory while interacting with the smart homecare control dashboard. Participants had to memorize steps to monitor and process alarms occurring in the homecare control dashboard.

First, in the planning phase, participants had to plan and memorize steps for processing alarms. Two stages were included in this phase. In stage one, the experimenter gave a table listing 10 alarm types and 23 steps, as shown in Table 1. Participants should match four out of twenty-three steps to each alarm type and put them in order based on their understandings. Stage one was designed to ensure that participants matched and ordered after thinking. Thus, participants were told that their answers should be logical and reasonable, although the answers did not link to their task performances. In stage two, the experimenter provided the recommended steps for memorizing. Besides, the experimenter would not bring back the answer sheet in stage one, enabling participants to compare. Both answer sheets would be taken back by the experimenter once the completion of this stage. Results of this pilot study indicated that 10 min were enough for participants to encode and memorize in this study environment. Hence, 10 min were required to complete this phase and 5 min were for each stage, respectively.

**TABLE 1:** A list of 10 alarm types and 23 steps.

Alarm types	Home security	Electricity
		Water
		Smoke
		Door sensor
		Infrared sensor
	Personal health	Heartbeat
		Blood pressure
		Blood sugar
		Bedridden
		Emotion
Steps	Check the condition of intelligent motor	
	Check the condition of the smart homecare sensor	
	Check the data again after 30 min	
	Check the data from infrared sensor	
	Check the location of the alarm	
	Check the location of the wearable device	
	Check if the mode of blood pressure monitoring is on or not	
	Check if the mode of blood sugar monitoring is on or not	
	Check if the mode of emotion monitoring is on or not	
	Check if the mode of heartbeat monitoring is on or not	
	Contact the nearby fire and rescue agency	
	Contact the nearby hospital	
	Create the form showing details of the alarm	
	Determine the medical priority of the patient	
	Inform the nurse who is in charge of the patient	
	None	
	Restart the smart home caring device	
	Save current data	
	Send a request for immediate house care services	
	Start the mode of continuous emotion monitoring	
	Turn on the indoor firefighting robot	
	Turn on the real-time video for monitoring	
	Turn on the vibration mode	

Second, the retention phase started after a 5-min break. A 10-min version attentional network test, CRSD-ANT (Weaver et al., 2013), was adopted. This attention task was conducted with the computer. Participants had to focus on fixation cross in the middle of the screen and tap with the arrow stimuli. This study employed this attention task to distract participants because prospective memory was likely to be impaired in real-life scenarios. Another 5-min break was given after this phase.

**FIGURE 1:**
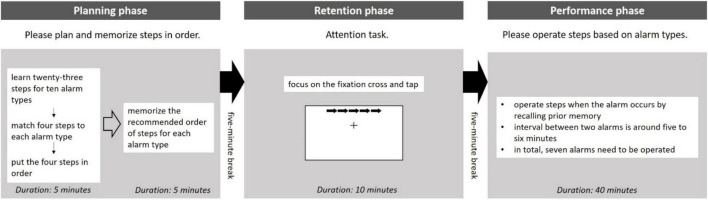
An overview of prospective memory task procedure.

Third, in the performance phase, participants had to interact with the smart homecare control dashboard. Participants would operate with the control dashboard when the two kinds of alarms were presented. The first one was an alarm that the machine itself could not handle. Participants should operate steps based on alarm types utilizing their prior memory in the planning phase. The prospective memory performance was calculated based on the accuracy of steps. Participants were reminded to process this alarm type accurately and rapidly. The second one was an alarm that the machine automatically had processed the alarm. A message box would pop up showing the information to inform participants. By clicking the “OK” button, participants could continue their task. The first alarm would occur every 5–6 min and the number of this kind of alarm in this phase was seven. Compared to the first alarm type, the occurrence of the second alarm type was more frequent, with an interval of around 30 s. To simulate the monitoring task in real workplaces in this phase, the experimenter told participants that both the kinds of alarms occurred randomly and no feedback was given before alarms. Specifically, every alarm was independent and participants had to concentrate on the smart homecare control dashboard and operate when needed. Approximately, 40 min were required for the completion of this phase. Figure 1 illustrates the whole prospective memory task procedure in this study.

After completing the performance phase, participants would fill out questionnaires, which measured the task load and boredom during the interaction in the performance phase. Around 2 h were needed to complete the whole experiment.

### Smart Homecare Control Dashboard

**FIGURE 2:**
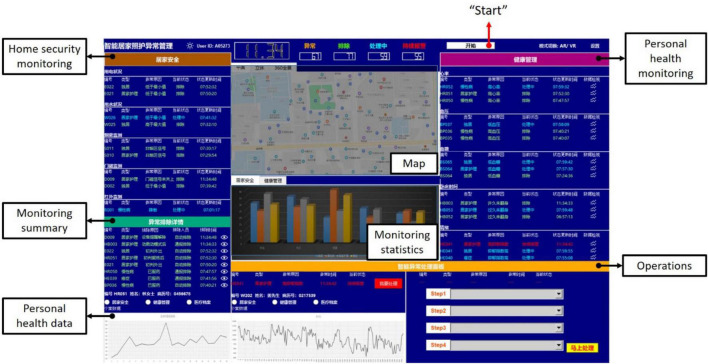
The interface of the smart homecare control dashboard.

The experimental platform was a smart homecare control dashboard developed with Python 3. As house care nurses, participants had to interact with this control dashboard for monitoring and operating when there were situations. Figure 2 shows the interface of the control dashboard consisting of a button to start the control dashboard, home security monitoring, personal health monitoring, monitoring summary, personal health data, operations, map, and monitoring statistics.

First, there was a white button at the top right, which participants had to click to start the control dashboard. Second, home security monitoring and personal health monitoring reported information about alarms. In home security monitoring and personal health monitoring, the alarm information would be presented with five parameters, namely, the alarming number, patient characteristic, reasons for the alarm, current alarm state, and update time of the alarm state. Besides, the word color changed as the current alarm states: the green was for those solved alarms, the blue was for those alarms were processing, and the red was for those alarms kept warning. The alarm summary displayed information of solved alarms with five parameters as well, while there were slightly different from the prior two displays. In addition to the alarming number, patient characteristics, and time of the alarm state update, reasons for solving the alarm and the way for solving the alarm (by human or machine) were reported. All the alarms were generated randomly and automatically by the control dashboard.

**FIGURE 3:**
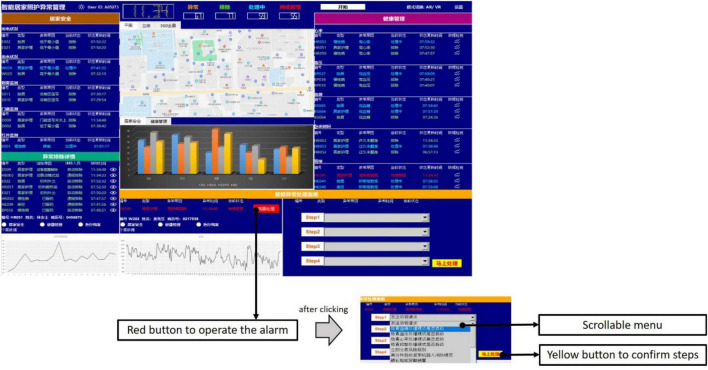
The alarm requiring participants to process in operations.

Third, the operations were located at the bottom left corner of the interface, designed to operate steps for alarms. The alarm that the machine could not handle would be presented in the left area of this panel. By clicking the red button, the information shown on the left would be transmitted to the top right area. Then, participants could process the alarm using the four scrollable menus in the right area. As shown in Figure 3, participants had to scroll to the corresponding step following the order of Step 1, Step 2, Step 3, and Step 4. The yellow button at the bottom right corner should be clicked to confirm the steps for processing. Then, a message box would pop up to inform participants that the control dashboard had been received successfully. By clicking the “OK” button, the control dashboard would continue. Unlike the alarm requiring human participants, the alarm that was directly handled by the machine would inform participants only with a message box. Again, a click on the “OK” button was required. All the alarms for processing were generated randomly and automatically by the control dashboard. In addition, the control dashboard would refresh once a new alarm occurred.

Next, the map was located at the top middle of the interface, presenting the location of the alarm immediately. In addition, historical health data of the patient were presented at the bottom left of the interface, which enabled a quick understanding of the condition of the patient. In addition, the number of alarms was reported with bar charts below the maps based on either home security monitoring or personal health monitoring.

### Electroencephalography Data Acquisition

Electroencephalographic data were recorded during the prospective memory task by Neuroscan 64-channels EEG system and SynAmps 2 amplifier system (bandpass = 0.05–100 Hz). The equipment was designed by Compumedics Limited, Australia. An online reference was placed on the left mastoid, and the average of the left and right mastoids was re-referenced offline. Data from the EEG were digitized at 1,000 Hz, and electrode impedance was below 10 kiloohms (kΩ). Electrodes placed at both the eyes (close to the temple) and the left eye (below and above) recorded horizontal and vertical electrooculographic (EOG) activity, respectively.

### Electroencephalography Preprocessing

Signal processing and analysis of EEG data were performed in Matlab R2021a (The MathWorks Incorporation, Natick, Massachusetts, United States) using the EEGLab toolbox (Delorme and Makeig, 2004). The data were filtered at 0.5–30 Hz after re-referencing. Channels with amplitudes exceeding ± 100 microvolts (μV) were marked as bad and excluded because their signals were noisy. In general, the threshold of bad channels was 15% of the total channels. This study ensured that the number of bad channels among all valid samples met the criteria. On average, 0.07 channels per participant (*SD* = 0.19) were removed. In addition, poor signals were removed visually before processing independent component analysis (ICA). According to the results of ICA, this study identified and removed components denoting artifacts using ICLabel (Pion-Tonachini et al., 2019). This study rejected components with higher rank by considering their IC labels, particularly for the labels of *Eye* with a probability of at least 0.5. On average, 13.22% (*SD* = 0.04) of components were excluded.

### Measures and Data Analysis

Subjective, objective, and physiological measures were collected in this study. First, this study used NASA-TLX (Hart and Staveland, 1988) and the Multidimensional State Boredom Scale (MSBS) (Fahlman et al., 2013) to examine task load and boredom subjectively. Both subjective measures were to ensure that the studied interaction with the smart homecare control dashboard matched the real-life scenarios, which of low task load and aroused boredom successfully.

Second, the objective measure was the prospective memory performance. This study calculated the accuracy of prospective memory based on steps that they submitted to process alarms during the performance phase. On average, the mean accuracy was 66.78% (*SD* = 0.19). In statistical analyses, the accuracy of prospective memory was a dependent variable.

Third, a Matlab function sub_stft, provided by Hu and Zhang (2019), was used for the time-frequency analysis. This study evaluated the brain activity of each region through the power within the frequency band of alpha (8–13 Hz). Seven brain regions were divided in this study. Table 2 presents the brain regions and their corresponding electrodes. Then, this study calculated the alpha power between the two time intervals in seven brain regions. One was a 10-min interval in the middle and the other was a 5-min interval before the end of the recording. Previous studies about boredom induction in vigilance and intelligent driving tasks suggested that 15–21 min were required to become bored (Martel et al., 2014; Samrose et al., 2020). This study then selected 15–25 min (a 10-min interval in the middle) after starting to ensure that participants were bored. In addition, the last 5 min were selected to make a comparison for exploring brain activities. This study compared alpha power differences by subtracting the alpha power in the middle interval from the alpha power in the latter interval. The positive value implied the inactive brain in the studied period. In contrast, the negative value indicated the activation of the brain region in this period. The alpha power differences between the two time intervals in seven brain regions were applied as independent variables in statistical analyses.

**TABLE 2:** The analysis of the brain regions and the corresponding electrodes in this study.

Brain region	Electrode
Anteriorfrontal (AF)	FP1, AF3, FPZ, FP2, AF4
Frontal (F)	F7, F5, F3, F1, FZ, F2, F4, F6, F8
Frontal central (FC)	FC5, FC3, FC1, FCZ, FC2, FC4, FC6
Centrotemporal (CT)	T7, C5, C3, C1, CZ, C2, C4, C6, T8
Centroparietal (CP)	CP5, CP3, CP1, CPZ, CP2, CP4, CP6
Parietotemporal (PT)	TP7, P7, P5, P3, P1, PZ, P2, P4, P6, P8, TP8
Parietooccipital (PO)	PO7, PO5, PO3, O1, POZ, OZ, PO4, PO6, PO8, O2
	

All the analyses were conducted with R Statistical Software (version 4.0.2). This study performed causal inference mediation analyses with the R package “*mediation*” (version 4.5.0). The default function *lm()* provided with the R environment was used for performing all the regression analyses.

## Results

### Subjective Results

Different boredom manipulations lead to diverse reactions and behaviors. According to the MSBS results, the experiment induced boredom through four paths: time perception (mean = 4.36, *SD* = 1.52), disengagement (mean = 3.95, *SD* = 1.00), inattention (mean = 3.86, *SD* = 1.23), and low arousal (mean = 3.69, *SD* = 1.05). In addition, participants received the system information passively. They were expected to implement memory tasks urgently during the experiment, in a way similar to air traffic control and memory. NASA-TLX (mean = 6.47, *SD* = 1.09) revealed that this study was of low task load (Grier, 2015). Overall, the four boredom dimensions and low task load guaranteed that this experimental scenario and task were comparable to the novel human–machine interaction.

In addition, this study conducted correlation analyses of MSBS, NASA-TLX, and prospective memory separately to reveal the relationship among them. First, the results of MSBS and prospective memory reported that the disengagement (*r*_*s*_ = 0.39,*p* = 0.04) and the total boredom score (*r*_*p*_ = 0.33,*p* = 0.08) were significantly correlated to prospective memory. Second, the results of NASA-TLX and prospective memory presented that the frustration level (*r*_*s*_ = −0.39,*p* = 0.04) was significantly correlated to prospective memory. The results of correlation analyses showed that both boredom and low task load correlated to prospective memory. Meanwhile, the results indicated that participants might attempt to focus on the task and get better performances.

### Research Question 1

To answer research question 1, this study conducted mediation analyses according to three criteria for determining the mediation effect proposed by Barron and Kenny (1986). At first, the direct effect was tested between the independent variable and the dependent variable. Next, the second analysis was to test the effect of the independent variable on the mediator. Then, this study tested the effect of the mediator and the independent variable on the dependent variable.

In the current analyses, the alpha power difference of anteriorfrontal (AF) was served as an independent variable, and prospective memory performance was used as a dependent variable. The alpha power differences of centroparietal (CP), centrotemporal (CT), parietotemporal (PT), and parietooccipital (PO) were analyzed as mediators.

Among the four mediators, the mediation effect was found in the alpha power difference of CP. Based on the CP, three regression models testing the mediation effect were presented as Model 1, Model 2, and Model 3.


Model 1 $$\text{Prospective memory}=\beta_{0}+c\times AF+e_{1}$$

Model 2 $$\text{CP}=\beta_{1}+a\times AF+e_{2}$$

Model 3 $$\text{Prospective memory}=\beta_{2}+c^{\prime }\times AF+b\times CP+e_{3}\;$$

According to model 1, results reported a significant direct effect of AF on prospective memory (*B* = 1.22,*p* = 0.046). Next, the results of model 2 showed a significant effect of AF on CP (*B* = 0.55,*p* = 0.075). Then, the results of model 3 also reported a significant effect of AF and CP on prospective memory (*B* = −76.57,*p* = 0.016). Afterward, this study determined whether the mediation effect is significant or not with R package “*mediation*” (Tingley et al., 2014). Non-parametric bootstrapping with 5,000 resamples was then conducted. The results revealed significant causal mediation effect (*B* = −53.1,*p* = 0.003), direct effect (B = −32.9,*p* = 0.038), and total effect (*B* = −86.0,*p* = 0.012). Table 3 summarizes the results of mediation analyses.

**TABLE 3:** Regression of prospective memory on AF and prospective memory.

Model	Results			
	Estimate	SE	*T*	*p*-value
**Model 1:**				
***Model information***				
Prospective memory on AF	1.22	0.58	2.13	0.046**
**Model 2:**				
***Model information***				
CP on AF	0.55	0.29	1.88	0.075*
**Model 3:**				
***Model information***				
Prospective memory on AF*CP	−76.57	27.37	−2.80	0.016**

**Non-parametric bootstrap confidence interval**	**Estimate**	**95% C.I.**		***p*-value**

Average causal mediation effect	−53.1	[−107.0, −11.7]		0.003**
Average direct effect	−32.9	[−75.9, −1.3]		0.038**
Total effect	−86.0	[−180.0, −14.2]		0.012**
Proportion mediated	0.6	[0.6, 0.9]		0.009**

N = 28; *p < 0.1; **p < 0.05.

**FIGURE 4:**
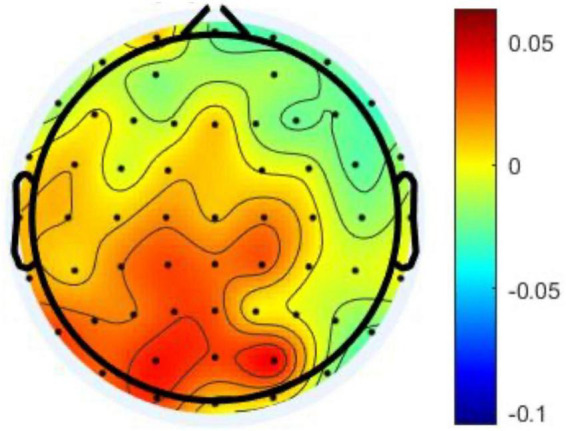
The topography demonstrates the differences of the alpha power between two studied intervals.

To sum up, this study found that the mediation effect of the alpha power differences in the CP was significant in the relationship between the AF and the prospective memory performance. In other words, the changes pf alpha power in CP could negatively influence the prospective memory. Moreover, Figure 4 provides further evidence that the prospective memory impairment resulting from the increased alpha power in CP was associated with the left hemisphere.

### Research Question 2

To answer research question 2, this study conducted analyses to figure out if there is any other brain activity that could impact the causal relationship between boredom and prospective memory. This study further included other variables (CT, PT, and PO) in three separate regression models to explore whether they could help interpret the relationship between alpha differences in the AF, F, and FC.

At first, this study constructed a regression model with the alpha power differences in the AF, F, and FC as independent variables and prospective memory performance as the dependent variable. Then, the CT, PT, and PO were introduced individually. According to the results, the PO could marginal significantly increase the prediction ability on prospective memory $\left(R_{AF,F,FC}^{2}=0.37,R_{AF,F,FC,PO}^{2}=0.45,p=0.06\right)$, while the CT and PT did not meet a statistically significant level. Furthermore, the results also revealed that the alpha power in AF, FC and F, and PO were nested. The results from the comparison of both models showed that the alpha power in the PO lobe could effectively enhance the predictability of boredom to prospective memory.

## Discussion

This study ensured that the experimental design focused on the features of future human–machine interaction scenarios, which were boredom and low task load, *via* a subjective questionnaire about the boredom and perceived task load during the interaction in the performance phase. Considering that alpha oscillations were found to be significant in CP and PO during the prospective memory task, the current findings will offer deeper indications in the following discussion.

### Alpha Oscillations in Centroparietal

Although the alpha power in the posterior brain regions is associated with feelings of relaxation and calm, it does not indicate that humanity is in the resting state (Lagopoulos et al., 2009). The parietal lobe is the common brain structure for novel thoughts (Schacter and Addis, 2007). Usually, creative and novel ideas require integration for many types of unrelated information and memory processes recalling past experiences. Benedek et al. (2018) conducted a study to investigate the brain mechanism of the generation of creative thoughts. Their fMRI results reported activation in the left inferior parietal lobe and supramarginal gyrus, which indicated that the mechanism for new idea construction was similar to the encoding and decoding of memory. In other words, it is not easy to generate creative ideas with merely either external information or memory. People acquire unrelated information based on their knowledge system. In addition, they retrieve semantic and episodic memory for divergent thinking and creative idea generation (Benedek et al., 2012). However, emotions, such as boredom, could lead to poor memory performance because daydreaming, mind-wandering, and other divergent thinking with high internal attention could worsen external stimuli perception for memory retrieval (Barron et al., 2011). Goldberg and Todman (2018) focused on boredom and studied mood-congruence memory. Their results pointed out that memory impairment might be associated with the encoding stage resulting from the attentional failure.

The dorsal and ventral attention networks are mainly responsible for top-down and bottom-up control, respectively. From the neuroscience perspective, the dorsal attention network relies on parietal activity and the ventral attention network links to the temporal lobe. Previous studies have shown that behaviors with top-down control are related to alpha oscillations in the parietal lobe, and they could hinder knowledge-based memory processes because of the limitation of attentional buffer capacity (Klimesch, 2012; Benedek et al., 2014a). Using top-down control to cope with memory-based tasks critically depends on alpha oscillations because large and distributed brain networks are related to the transfer of information between memory systems (Min and Park, 2010). To prevent mind-wandering and distraction, frequent shifting between both attention networks can be observed accordingly. Vossel et al. (2009) further revealed that the activation of the dorsal and ventral attention networks was a representation of the reorienting processes for regaining attention.

This study was conducted by performing a bottom-up and memory-based experiment. The significant effect of the parietal lobe might indicate that participants in the present intelligent interaction could lead to the loss of situation awareness similar to the top-down processing. Benedek et al. (2014b) investigated the alpha power in the parietal regions based on hemispheres. Their results suggested that higher internal attention was related to higher alpha power in the right-parietal lobe. Meyer et al. (2018) indicated that hemispheric differences played an important role when it comes to internal and external attention. In this study, Figure 4 presents that the majority of the increase of alpha power were in the left-posterior brain regions. This study suggested that the boredom coping strategy while interacting with intelligent systems should be related to the unstable attention mechanism.

The findings in the study of Yakobi et al. (2021) might help to interpret the results of this study. They argued that people continued in sensory processing even though they were asked to do tasks with low task load in an uninteresting environment. Indeed, continuous sensory processing could imply the inattentive status of individuals, making people not sensitive enough to detect the cues of prospective memory retrieval. Moreover, according to the prospective memory decision control (PMDC) model (Stirckland et al., 2018), Boag et al. (2019) reported that prospective memory could facilitate ongoing decision-making when there was no time pressure. In contrast, time pressure would occupy limited-capacity cognitive resources and reduce attentional control. Overall, the practical implication of this study for preventing prospective memory impairment is suggested for passive user interaction, i.e., future task allocation in human–machine collaborative intelligent systems should concentrate on operators’ attention management and inhibit top-down control and divergent thinking. The meaning and attentional components (MACs) model can further support the proposed practical implication since the boredom state occurring in most human–machine collaboration cases is regarded as attentional boredom resulting from understimulation (Westgate and Wilson, 2018).

### Alpha Oscillations in Parietooccipital

Introducing parietooccipital activity can significantly predict prospective memory that could be influenced by boredom. Although boredom is associated with alpha oscillations, especially in the frontal lobe, this study further found that the parietooccipital lobe might be critical for investigating prospective memory, which would decline with boredom. As discussed in section “Alpha Oscillations in Centroparietal,” the activation of the parietal lobe is linked to creativity and divergent thinking. According to this study, the functional connectivity between the parietal and occipital lobes should be worth noting regarding the relationship between boredom and prospective memory. This study referred to the empirical research and gathered two reasons for the results. First, the default mode network (DMN) should be activated in the current boredom induction scenario, which is a brain activity to cope with boredom induced by low task load. According to the study of Fink et al. (2018), the time-related changes in divergent thinking during the creativity task were related to the left supramarginal gyrus and the right occipital lobe. In addition, Shi et al. (2018) then reported causality between the DMN and creativity. This study argued that brain activity in the PO might reflect internal brain conditions even though the EEG evidence lacked spatial resolution. The DMN is evoked when one is experiencing disengagement and inattention (Danckert and Merrifield, 2018). Meanwhile, from a psychophysiological perspective, the DMN indicates the resting brain and unoccupied brain regions (Manson et al., 2007). Second, visual processing was engaged in the experiment, as participants had to keep their eyes open to complete the task, increasing the importance of the occipital lobe. The occipital lobe is the primary visual processing center, and alpha power activity in the occipital lobe could be associated with the facilitation of visual information selection, including conscious execution (Jensen et al., 2002; Samaha et al., 2015).

After the discussion about the oscillation in PO, the question about how the PO can help predict prospective memory during boredom will be explained accordingly. Park et al. (2011) investigated the functions of working memory and visual-related brain activity. Their findings suggested that blind people presented effective connectivity from the DMN to the left frontoparietal network and from the occipital cortex to the right frontoparietal network during the 2-back task. The experiments in their study did not cover the visual tasks. Instead, they applied verbal and sound cues with tone and spatial information for the investigation. The research team further suggested that the occipital cortex was engaged in the execution of working memory. Moreover, Smith et al. (2011) revealed that working memory was closely associated with prospective memory. Specifically, people who have a larger working memory span could probably have higher prospective memory performance. Therefore, this study reports that brain activity in PO could demonstrate memory mechanisms, which should be adapted to the memory processes with most sensory channels.

However, dealing with visual information, such as cues, is crucial for prospective memory retrieval and decision-making in real-life settings. The alpha oscillation in PO is utilized for the anticipatory neural biasing mechanism preparing for visuospatial attention and motor function and maximizing their reward of decision-making (Heuer et al., 2017). This study then suggests that using alpha oscillations in PO to understand prospective memory during boredom should be applied to both psychophysiological and practical areas.

### Limitations and Future Research

There are some limitations to this study. First, the induction of interacting boredom extended the time length of the EEG experiment, which might have caused fatigue and other negative effects. However, this study did not analyze other emotions toward perspective memory separately. Second, this study is limited by the small sample size due to the long experimental duration.

Although the results of this study are helpful to identify the link between boredom and prospective memory, they are preliminary and await further replication for concise interpretation in future research. First, future studies need to look at the effects on top-down and bottom-up information processing differences since this study did not directly assess their influences and the exact mechanisms remain unknown. Second, this study revealed the DMN to provide reasonable explanations of the relationship between boredom and prospective memory, and further investigation is needed to support this argument. Third, this study has concluded the relationships from the signals from the scalp, but it is worth noting that the inferior brain regions, such as the inferior frontal gyrus, could also be contributing to the influences of boredom on prospective memory. Thus, this study proposes that further evidence, based on a higher spatial resolution, would be beneficial to understanding the link between boredom and prospective memory.

## Conclusion

This study explored the effects of boredom on prospective memory. The alpha power in seven brain regions while interacting with the intelligent system was measured with EEG. Alpha oscillations were used to examine the relationship between boredom and prospective memory.

The results of this study reported that the prospective memory impairment was associated with distraction while bored in interacting with the intelligent and complex system. The results showed that alpha oscillations in both the parietal and parietooccipital regions supported the proposed findings. The mediation effect on the parietal region pointed out that the changes pf alpha power in CP could negatively influence the prospective memory. In addition, the finding from the parietooccipital activity was associated with the DMN, which revealed that individuals would consciously avoid irrelevant external information when they were bored in the study context. Therefore, the findings from this study suggest that attention management and influences of processing visual information could improve the preparation of prospective memory and enhance decision quality during the task periods.

The increasing passive user interaction with intelligent systems shapes novel human–machine task allocation styles, but human workers encounter challenges to handle situations needing human control effectively. This study used EEG to enumerate the way that boredom influences prospective memory. Consequently, this study provided evidence from brain activity to understand behavioral changes in human–machine intelligent interactions. Furthermore, the findings of this study could be applied to the foundation of future human-centered system design and intervention measures.

## Data Availability Statement

The raw data supporting the conclusions of this article will be made available by the authors, without undue reservation.

## Ethics Statement

The studies involving human participants were reviewed and approved by the Department of Industrial Engineering, Tsinghua University. The patients/participants provided their written informed consent to participate in this study.

## Author Contributions

P-HC and P-LR: conception and design of the work, data analysis, and interpretation. P-HC: manuscript. P-LR: validation and supervise this study. All authors read and approved the final manuscript.

## Conflict of Interest

The authors declare that the research was conducted in the absence of any commercial or financial relationships that could be construed as a potential conflict of interest.

## Publisher’s Note

All claims expressed in this article are solely those of the authors and do not necessarily represent those of their affiliated organizations, or those of the publisher, the editors and the reviewers. Any product that may be evaluated in this article, or claim that may be made by its manufacturer, is not guaranteed or endorsed by the publisher.
